# Unusual histological presentation in neurofibromas: Two case reports

**DOI:** 10.1186/1757-1626-1-188

**Published:** 2008-09-29

**Authors:** Deepti Joshi, Nitin Gangane, Sanjeev Kishore, Sunita Vagha

**Affiliations:** 1 Department of Pathology, Mahatma Gandhi Institute of Medical Sciences, Sevagram, Maharashtra, India; 2 Department of Pathology, Himalayan Institute of Medical Sciences, Dehradun, Uttaranchal, India; 3 Department of Pathology, Jawaharlal Nehru Medical College, Sawangi Meghe Maharashtra, India

## Abstract

Various histological variants of neurofibroma have been described. The objective of this paper is to discuss the unusual histological findings seen in two cases of neurofibromas associated with neurofibromatosis type 1 Both cases presented with multiple subcutaneous nodules. Surgical excision of the largest nodule was done in both the cases. Histological examination of case no.1 revealed a benign tumor of the peripheral nerve sheath, of neurofibroma type with presence of mucus producing glands. The epithelial component was benign in this case. The second case showed presence of rosettes in between areas of typical neurofibroma.

## Background

Neurofibromatosis-Type1 (von Reckling hausen's disease) presents with various pathological manifestations like histological abnormalities of epidermis (café au lait spots), iris (Lisch nodules), skeleton (malformations), blood vessels (mesodermal vascular dysplasias, brain (glial tumors and hamartomas), intestine (endocrine and gastrointestinal stromal tumors) and the peripheral nerve sheath (neurofibromas and malignant peripheral nerve sheath tumors) [1]. Neurofibroma is a benign peripheral nerve sheath tumor with distinctive histological features. Various distinct histological variants of neurofibroma have been described. We hereby report the unusual histological presentation of neurofibroma seen in two patients with NF Type 1.

## Case presentation

### Case number 1

A 35 year old male patient presented with multiple, nodular swellings over back (in the region of thoracic spine) and thigh. Freckles were noted in axillary region and groin. Patient's mother also had nodular swellings though none were biopsied. Swelling over thigh was excised and submitted for histopathology. Gross examination revealed presence of a 4 × 2.5 × 1 cm grey white tumor which was mucoid in consistency.

**Figure 1:**
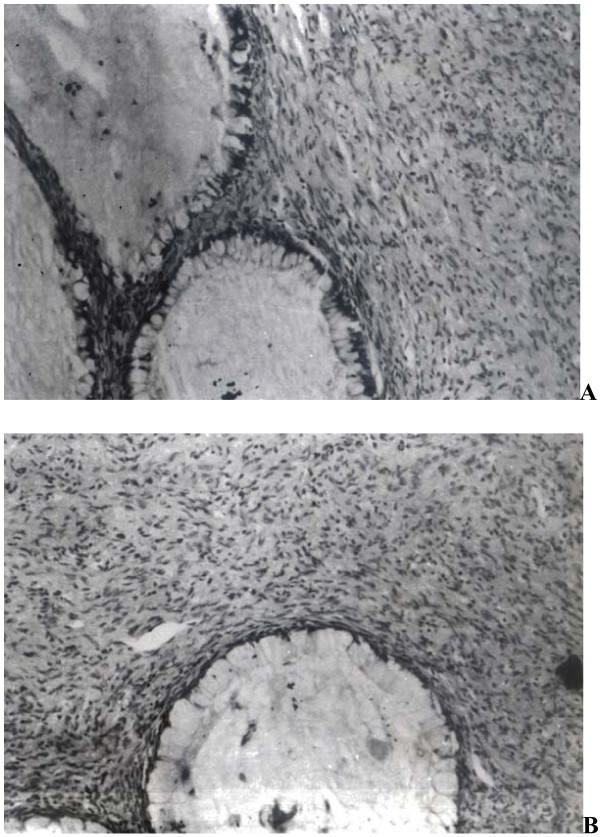
**(a): Section shows slender spindle shaped cells embedded in a collagenous stroma along with presence of mucus producing glands.** (H&E ×100) (b): Glands are lined by goblet cells and show presence of intraluminal mucin. (H&E ×400).

Microscopic examination showed presence of tumor consisting of spindle cells having slender, curved, often wavy nuclei embedded in a collagenous stroma. Cytoplasm of these cells was indistinct and nuclear pleomorphism or mitotic figures were not present. These cells were also seen arranged in interlacing bundles but no nuclear palisading or Antoni A/B areas were demonstrated. Tumor also showed presence of mucus producing glands lined by a single layer of benign tall columnar epithelial cells with basally located nuclei and abundant clear cytoplasm (Figure 1). A diagnosis of benign glandular peripheral nerve sheath tumor (PNST or glandular neurofibroma) was given.

### Case number 2

A 32 year old male patient presented with swellings over neck, thigh and upper extremity. The patient had distinctive skeletal abnormalities and careful clinical examination revealed presence of café au lait spots in left posterior axillary fold. Swelling over neck was excised and submitted for histopathology. It measured 3 × 1.5 × 1 cm and the cut surface was homogenously tan grey and glistening.

**Figure 2:**
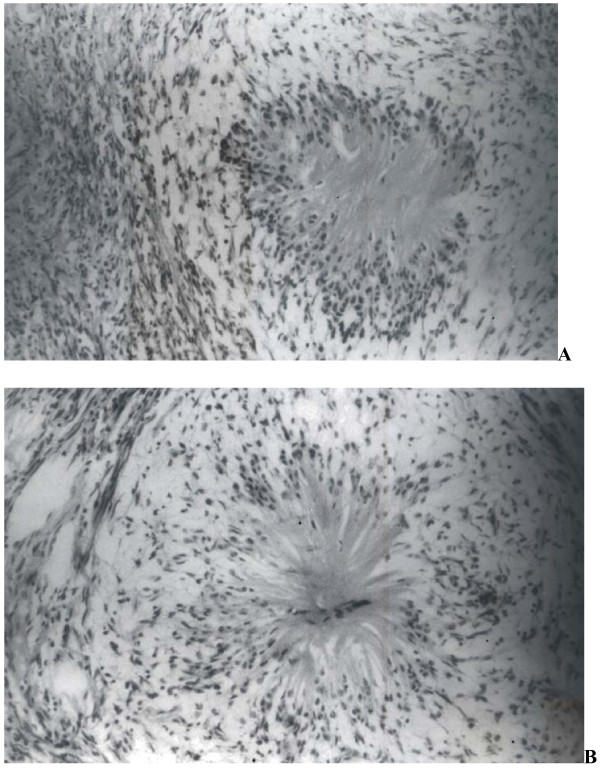
**(a): Section shows presence of rosette like structures between areas of typical neurofibroma.** Rosettes display central eosinophilic, fibrillary core and peripheral palisading by neuronal cells. (H&E ×100) (b): Another rosette showing presence of central capillary in the fibrillary core. (H&E ×400).

On microscopic examination, variably spaced slender, spindle shaped cells were seen in a loose, myxoid and collagenous stroma. No mitosis was observed in this spindle cell component. Rosette like structures were seen amidst these areas of typical neurofibroma. These rosettes displayed presence of a central, eosinophilic, fibrillary core which was surrounded by round to elongated cells. The cells bordering the rosettes were seen merging into the adjacent stroma (Figure 2). The tumor was diagnosed as neurofibroma and the presence of focal rosette like structures was noted.

## Discussion

First described by Garre et al [2]. in 1892, glandular differentiation is the rarest form of divergent differentiation seen in peripheral nerve sheath tumors (PNST) which also includes cartilage, bone, chondrosarcoma, osteosarcoma and rhabdomyosarcoma. Around 40 cases have been reported so far in world literature. Diagnostic criteria includes evidence supporting a finding of nerve sheath tumor, presence of true glandular epithelium, not entrapped glands or pseudoepithelium. The vast majority of PNST harbouring these glands have been malignant peripheral nerve sheath tumor (MPNST) [3]. The glandular epithelium is usually benign though there are sporadic case reports of PNST with malignant glandular component [4].

Glandular neurofibromas are rare and in an extensive review by Woodruff and Christenstein, only 2 out of 25 analyzable cases of glandular peripheral nerve sheath tumors were neurofibromas. Both of these cases occurred in young females, were associated with NF Type 1 and were finally diagnosed as plexiform neurofibromas [3]. No history of neurofibromatosis was present in the other reported cases of glandular neurofibromas [5-8].

Most important differential diagnostic consideration in a case of glandular neurofibroma is schwannoma with entrapped adnexal structures. Schwannomas usually display typical Antoni A and B areas along with presence of Verocay bodies. Trapped adnexal glands are generally seen in clusters and are connected to each other. In the absence of typical morphological features, differentiation between these entities may be difficult and immunohistochemistry (IHC) may be employed. Glands in true glandular PNST are devoid of myoepithelial cell lining and are hence non reactive for muscle common actin (HHF-35). This glandular epithelium is not only reactive for cytokeratins but also for neuroendocrine cell markers (chromogranin, serotonin and somatostatin). Entrapped adnexal glands in schwannoma show the presence of myoepithelial cell layer and are non reactive for neural markers. Stromal cells show S-100 positivity in both the cases [3].

Though IHC was not employed in our case (case number 1), the tumor was diagnosed as neurofibroma as characteristic features of neurofibroma (spindle cells with short, curved nuclei embedded in a collagenous stroma) were seen on histology sections. Moreover, history of NF-type 1 in the patient corroborated the diagnosis of neurofibroma. The glands seen in case number 1 resembled mucus secreting glands rather than eccrine glands of skin adnexa.

It is interesting to note that adnexal glands have also been reported in neurofibroma and it has been proposed that the tumor appeared in the nerves around the eccrine glands and grown to the subcutaneous tissue, and the glands might have been left behind rather than entrapped by the growing tumor [9].

The histogenesis of glandular PNST is still not clear and it may be attributed to the metaplastic potential of Schwann cells or the presence of primitive neural crest cells that migrate with Schwann cells along the peripheral nerves [4].

Neurofibroma with rosette like structures is exceedingly rare. Enzinger reviewed a unique case of neurofibroma showing presence of mucus secreting glands and focal rosettes [10]. A recently described variant of neurofibroma with rosettes is dendritic cell neurofibroma with pseudorosettes (DCNP) [11]. This tumor is seen in adults and has been described mostly in patients without a history of NF-1, [11,12]. though cases arising in association with NF-1 have also been described [13]. The lesion is well circumscribed and occurs in superficial dermis of head, trunk and extremities. It is comprised of two types of cells. Type 1 cells are small lymphocyte like cells with slightly cleaved nuclei which are concentrically arranged around larger type 2 cells having vesicular nuclei and copious cytoplasm. These type 2 cells have dendritic extensions which form the core of these pseudorosettes. On IHC, type 2 cells and most type 1 cells stain for CD57 and S-100 [11].

Another important differential diagnostic consideration is Schwannoma with neuroblastoma like rosettes. In this tumor, small round to oval cells are layered around a central eosinophilic fibrillary material. These cells may also show presence of intranuclear cytoplasmic inclusions [14].

Other spindle cell tumors which may show presence of rosettes are Low grade fibromyxoid sarcoma (LGFMS) and the closely related Hyalinising spindle cell tumor with giant rosettes (HSCT). These tumors are comprised of fusiform or spindled cells enmeshed in a heavily collagenized stroma showing abrupt transitions to myxoid zones. Mitotic activity is typically low. Cases diagnosed as "hyalinizing spindle cell tumor with giant rosettes" contain a variable number of collagen rosettes [15]. though rosettes are also seen in upto 40% of cases of LGFMS [14]. Interestingly, LGFMS like areas have been described in relation with low grade MPNST [16]. But both LGFMS and HSCT are tumors of fibroblasts rather than schwann cells and show much weaker expression of S-100 protein [14]. The stroma of case number 2 was Shwannian and it lacked the presence of fibroblastic cells arranged in whorls and curvilinear blood vessels which are the characteristic features of LGFMS,[14]. hence the tumor was suspected to be neurofibroma.

## Conclusion

Glandular benign PNST and neurofibromas with rosette like structures are extremely rare and reflect the histological diversity seen in PNST. It is important to document these entities as these need to be differentiated from other potentially malignant tumors.

## Abbreviations

NF: Neurofibromatosis 1; PNST: Peripheral nerve sheath tumor; MPNST: Malignant peripheral nerve sheath tumor; IHC: Immunohistochemistry; DCNP: Dendritic cell neurofibroma with pseudorosettes; LGFMS: Low grade fibromyxoid sarcoma; HSCT: Hyalinising spindle cell tumor with giant rosettes.

## Competing interests

The authors declare that they have no competing interests.

## Authors' contributions

DJ confirmed the diagnosis of the cases, and wrote the first draft of the manuscript. NG confirmed the diagnosis of the cases, and critically reviewed the manuscript. SK reviewed the case slides of case number 1 and verified the diagnosis. SV reviewed the case slides of case number 2 and verified the diagnosis.

## Consent

A written consent has been obtained from both patients. No personal identifiers are used in this case report.
